# Analysis of Occupational Infections among Health Care Workers in Limpopo Province of South Africa

**DOI:** 10.5539/gjhs.v5n1p44

**Published:** 2012-11-02

**Authors:** Ntambwe Malangu, Adelaide Legothoane

**Affiliations:** 1 Department of Epidemiology and Biostatistics, University of Limpopo, Medunsa Campus, South Africa

**Keywords:** occupational, hospital acquired infections, South Africa

## Abstract

**Objective:**

Occupational infections particularly hospital-acquired infections (HAIs) are a serious problem in the healthcare industry worldwide. This study purported to investigate their prevalence and risk factors among healthcare workers from Limpopo province of South Africa.

**Methods:**

Cases about occupational infectious diseases of healthcare workers from Limpopo province that were submitted to the Compensation Commissioner from January 2006 to December 2009 were reviewed.

**Results:**

The total number of cases of infectious diseases reported during the study period was 56; of these, 83.9% (47) of cases were for tuberculosis, 10.7% (6) for cholera, and 5.4% (3) for chickenpox. Nurses were the most affected. Risk factors associated with the acquisition of infection diseases were as follows. The majority of those infected were female (67.9%), aged over 40 years (57.1%), and who had worked for over 10 years (59.2%). With regard to length of time it took for one to be infected, overall it took 13.6±9.7 years from the year of employment to being infected. This duration was just 5.7±4.2 years in HCWs younger than 40 years versus 18.4±9.0 years in those 40 years and over (p=0.001); and 11.4±10.3 years in nurses versus 17.1±7.8 years in non-professional staff members (p=0.046). Mopani district, situated in a rural setting was the most affected as 24 of the 47 cases of tuberculosis occurred there.

**Conclusion:**

In conclusion, the most common occupational infection or hospital acquired infection among healthcare workers in Limpopo province of South Africa was tuberculosis. It infected mainly nurses from the rural health district of Mopani. Younger age and being a nurse were significant risk factors associated with being infected early.

## 1. Introduction

Occupational infections particularly hospital-acquired infections (HAIs) are a serious problem in the healthcare services worldwide. They represent a risk to both patients and healthcare workers (HCWs). It is estimated that between 5% and 10% of patients admitted to acute care hospitals acquire at least one infection and over the last decades the incidence has been documented to be increasing particularly in the United States and Europe (Eriksen et al., 2005; Hopmans et al., 2007; Klevens et al., 2007; Pittet et al., 2008).

However data on HAIs from developing countries are scare. In Southern Africa, published reports were found for Malawi where 3.6% to 6% of HCWs have been reported to be infected with tuberculosis (Harries et al., 1999; Kanyerere et al., 2003). Similar reports on the prevalence of tuberculosis and other infections among healthcare workers in South Africa were not found, but data from a systematic review suggest that in developing countries, rates of latent tuberculosis infections ranges from 0.5% to 14.3% (Joshi et al., 2006).

Given the lack of published data on the extent of hospital acquired infections in health care settings in South Africa, this study purported to investigate their prevalence and risk factors among healthcare workers from Limpopo province of South Africa based on data submitted to the Compensation Commissioner in terms of the Compensation for Occupational Injuries and Diseases Amendment Act 61 of 1997 (National Department of Health [NDOH], 2011). In doing so, this study endeavoured to contribute to raise awareness on this topic and provide data that could be used for planning appropriate interventions.

## 2. Materials and Methods

Data submitted to the Compensation Commissioner from January 2007 to December 2009 were extracted using a pre-designed data collection form. This form was designed by the investigator for this study. Cases were defined as reports about any healthcare-acquired infection as submitted for compensation by healthcare workers of Limpopo province. This province is home to 11% of the 50.6 million South African populations (Statistics South Africa, 2012); it is divided into five municipal districts, namely, Capricorn, Mopani, Sekhukhune, Vhembe and Waterberg.

The Capricorn district is named after the Tropic of Capricorn, which passes through this northern part of the province. It is the area of the province that is in close proximity to the neighbouring countries of Botswana, Zimbabwe and Mozambique. It is a semi-urban area as it includes major towns such as Polokwane and large farms. Mopani district includes the Great Olifants River that runs through the Kruger National Park forming the southern border of the province. Sekhukhune province is named after the late King Sekhukhune; it is a rural province whose economy is based on mining and agriculture. Vhembe district is named after the impressive mountain range that stretches from the west to the east of the region. It is rural area boasting of several ancient artworks on rocks. Waterberg is situated in the magnificent Waterberg Mountains range. It is also a rural area (Statistics South Africa, 2012). Ethics approval to conduct this study was obtained from the Medunsa Research Ethics Committee.

The following data were collected: age and gender of victim, year of report, year of employment, professional category, the name of the infection and the health district. From the year of employment, the number of years of working experience and the time it took to being infected were calculated. Data were coded and captured in a spreadsheet and the accuracy of capturing was checked by means of a comparison of a printout to the original forms. The age was dichotomised into less than 40 years and 40 years and above based on the mean value of the age parameter. Analysis of variance was conducted to test the difference between younger and older victims with regard to how long it took for one to be infected. The level of statistical significance was set at p<0.05.

## 3. Results

**Figure 1:**
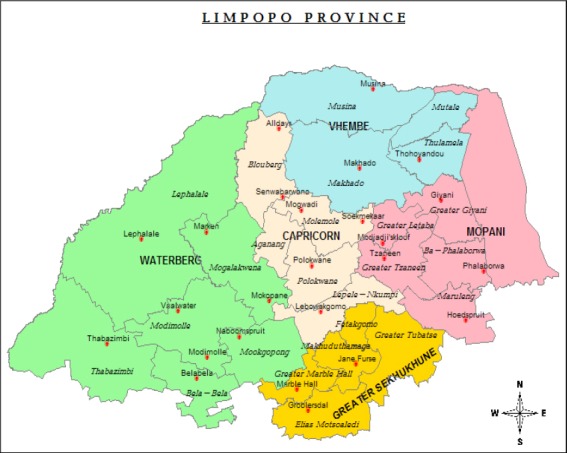
Map showing the health districts of Limpopo Province in 2009

**Figure 2:**
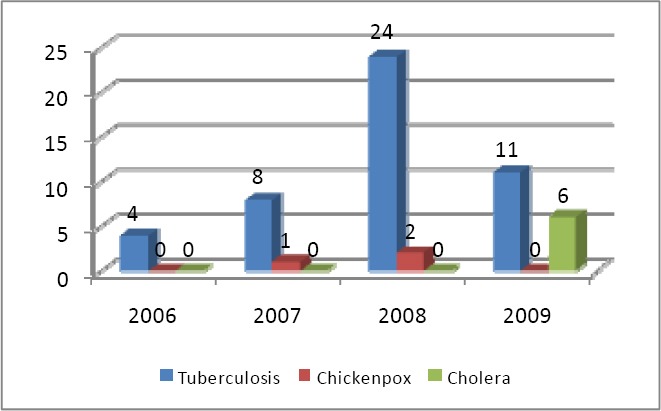
Cases of hospital acquired infections per year in Limpopo Province of South Africa 2006-2009 (n=56)

The total number of cases of infectious diseases reported during the study period was 56 in the five health districts (Figure 1); of these, 83.9% (47) of cases were for tuberculosis, 10.7% (6) for cholera, and 5.4% (3) for chickenpox. Tuberculosis was reported every year during the study period; while cholera was reported only in 2009; chickenpox occurred in 2007 and 2008 (Figure 2).

The ages of the victims ranged from 24 to 63years, with a mean of 42.7± 9.5years; their median age was 45years. The most affected professional category was health professionals, namely nurses (Table 1). While only one female medical doctor suffered from tuberculosis, all three cases of chickenpox, 63.8% (30 of 47) of tuberculosis, and two of the 6 cases of cholera affected nurses. Among non-professional staff, the cleaning personnel was the most affected as 17% (8 out 47) of tuberculosis cases and four of the six cases of cholera occurred among them. In addition, other categories of staff members were also infected by tuberculosis including administrative and maintenance staff (Table 2).

**Table 1:** Characteristics of staff members infected in Limpopo Province of South Africa 2006-2009 (n=56)

Characteristics	Frequency	Percent
**Professional category**		
Nursing staff	35	62.5
Cleaning staff	12	21.4
Administrative clerks	4	7.1
Kitchen staff	2	3.6
Medical staff	1	1.8
Drivers	1	1.8
Maintenance staff	1	1.8
**Sex**		
Female	38	67.9
Male	18	32.1
**Age category**		
40years and above	32	57.1
Under 40years	24	42.9
**Years of experience**		
Over 10years	29	59.2
Up to 10years	20	40.8
**Health district**		
Mopani	24	42.9
Vhembe	11	19.6
Waterberg	10	17.9
Sekhukhune	9	16.1
Capricorn	2	3.6

**Table 2:** Categories of personnel affected by tuberculosis in Limpopo Province of South Africa 2006-2009 (n=47)

Professional category	Frequency	Percent
Nurses	30	63.8
Cleaning staff	8	17.0
Administrative clerks	4	8.5
Kitchen staff	2	4.3
Drivers	1	2.1
Maintenance staff	1	2.1
Medical doctors	1	2.1
Total	47	100.0

Other factors associated with the acquisition of infectious diseases were the number of years of working experience, the age and the sex of the healthcare worker as well as the working environment, particularly the geographical location. Although not statistically significant, the majority of those infected were female (67.9%), aged over 40 years (57.1%), and who had over 10years of work experience (59.2%).

With regard to the length of time it took for one to be infected, overall it took 13.6±9.7years from the year of employment to being infected; ranging from 1 to 36years. There was a statistically significant difference between younger and older HCWs; it took on average 5.7±4.2years for those younger than 40years versus 18.4±9.0years in those 40years and over (p=0.001). A similar observation was made when comparing nurses versus non-professional staff members; nurses were infected earlier than the non-professional HCWs (means of 11.4±10.3years versus 17.1±7.8years, p=0.046).

**Figure 3:**
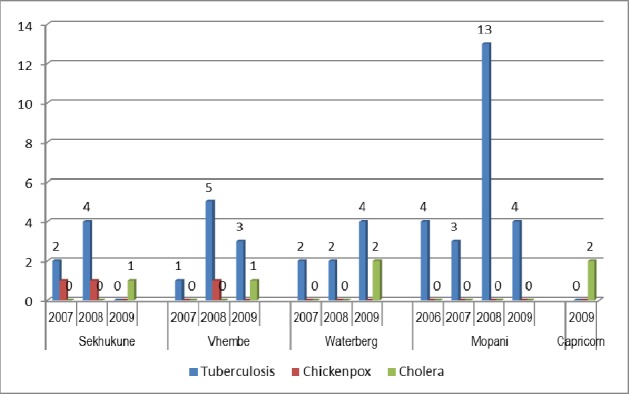
Cases of infectious diseases per district in Limpopo Province of South Africa 2006-2009 (n=56)

**Figure 4:**
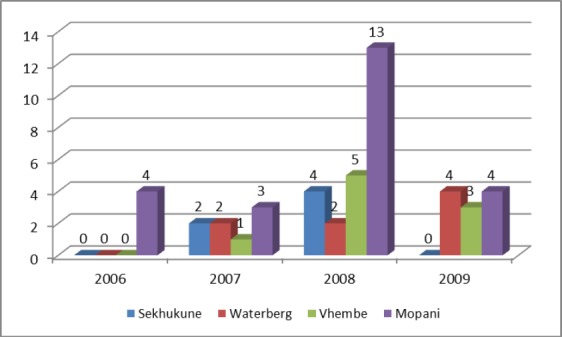
Cases of tuberculosis per year and district in Limpopo Province of South Africa 2006-2009 (n=47)

There were notable differences among health districts with regard to the distribution of infections. Cholera was reported in four of the five districts, except in Mopani. In two districts, Sekhukhune and Vhembe, three infectious diseases were reported, namely, chickenpox, cholera and tuberculosis. Mopani district, situated in a rural setting was the most affected by tuberculosis as 24 of the 47 reported cases occurred there. In contrast, in the urban district of Capricorn, no single case of tuberculosis or chickenpox was reported, but 2 of the six cases of cholera occurred there (Figure 3). Moreover, from 2007 to 2009, tuberculosis cases have been consistently reported in three districts of Waterberg, Sekhukhune, and Mopani (Figure 4).

## 4. Discussion

This study found that the most common hospital-acquired infection that affected healthcare workers in Limpopo province was tuberculosis; it was responsible for 83.9% of all cases reported to the Compensation Commissioner. During the study period, based on average number of nurses the province, the 30 cases of tuberculosis among nurses represent an annual incidence of 204.7 or 205 cases per 100,000 nurses (Health System Trust, 2011). This finding is consistent with reports from other settings that show that healthcare workers are a risk of acquiring tuberculosis and other diseases such as chickenpox and cholera from their workplaces (Sepkowitz, 1996; Joshi et al., 2006; Menzies et al., 2007; Drobniewski et al., 2007; Rebman et al., 2008).

The findings from this study show that cholera infected healthcare workers only in 2009 in four health districts. Although this finding is consistent with the documented cholera outbreak among patients from November 2008 to January 2009, it suggests also that the outbreak may have been not adequately handled as it spread across districts and resulted in its transmission to healthcare workers (NDOH, 2009). Moreover, the findings from this study demonstrate that the infection control measures that were implemented in four health districts during the study period did not seem to have been effective as cases of acquired infections of tuberculosis continued to occur every year particularly in Mopani district. The above remarks concur with previous findings by Yassi and co-workers (2009) who reported that there were weaknesses in the use of N95 respirators and safe handling of sharp instruments as well as inadequate access to supplies and personal protective equipment among HCWs they had interviewed. However, in the Capricorn district, there seems to be some effective tuberculosis infection control measures in place as no cases of tuberculosis or chickenpox were reported there during the study period.

With regard to risk factors, namely age and gender, the fact that the majority of those affected were female and older workers is consistent with the distribution of these groupings in the workforce. This finding is similar to a report from Nigeria where female HCWs were also the most affected (Salami & Oluboyo, 2008). In addition, the findings of this study show also that nurses, and particularly those younger than 40years old were at a greater risk of being infected early. Studies from other settings have reported also similar findings. In Nigeria, the duration from employment to being infected varied from half a year to 11.5years; while in Morocco, it has been shown that the annual incidence rates of tuberculosis is ten times higher in HCWs who work with patients infected with tuberculosis than in the general population (Salami & Oluboyo, 2008; Yasi et al., 2009).

In comparison to developed countries where latent tuberculosis infection (LTBI) has been assessed, age has been found as one of the risk factors (Schablon et al., 2009). In addition, Menzies and colleagues (2007) cited occupational risk factors such as working in internal or respiratory medicine wards, and extensive years of work in healthcare; while De Vries and co-workers (2006) reported that delayed diagnosis of tuberculosis in older patients was the main cause of transmission of tuberculosis from patients to HCW in the Netherlands. These reports concur with the findings of this study and other reports from other developing countries cited above with regard to the influence of age.

The findings of this study are to be interpreted taking into account the limitations inherent to the design of this study; as a retrospective study, no temporal link could be established between variables; some data could not be collated such as the types of tuberculosis; whether treatment for tuberculosis was offered and its outcomes; whether the HCWs were successful in getting compensated or not; as well as the details of infection control measures implemented within the health districts. Moreover, because the study was based on compensation data, it is likely that all cases of occupationally-acquired infections may not have been diagnosed as such and reported; hence, the actual incidence could be greater.

Despite the above limitations, the findings from this study have several implications as explained below. As suggested by Harries and colleagues (2010), we also advocate a system-thinking approach including the following elements.

As a matter of policy, there is a need for a review of the infection control measures implemented in the province so that gaps could be identified and appropriate corrective actions and interventions could be implemented. With regard to practices, the consistent reoccurrence of tuberculosis and the occurrence of chickenpox cases suggest that screening measures should be in place in order to detect cases timely among HCWs and provide adequate preventive and curative treatments. In particular, measures should be put in place to address the following vaccine-preventable infections: hepatitis B, influenza, measles, mumps, rubella, and varicella. The findings of this study suggest also that it is important that efforts be put into the implementation of the guidelines for the prevention of tuberculosis in the healthcare settings (WHO, 2009). These include implementing appropriate patient risk assessment measures, and instituting an active hospital tuberculosis case surveillance and developing an effective institutional infection control plan based on the local infrastructure.

Because diverse categories of healthcare personnel were infected, this observation implies that infection control measures should be implemented in a system-wide fashion rather than focussing on hospital wards and front-line healthcare workers, nursing and medical staff members. In particular, the finding that cleaning and administrative staff members were among the most affected by tuberculosis, suggests that capacity building activities including training, provision of personal protective apparels, and implementation of environmental controls should involve all categories of personnel and all buildings where interactions with infected or suspected patients occur. Findings from this study suggest also that younger HCWs should be targeted with training in infection control as they seem to become infected quickly.

With regard to further research, this study suffered from some limitations inherent to its design. Because it was based on the review of records, the completeness of relevant data could not be guaranteed. In addition, it is plausible that claims for other compensable and blood-borne infections such as hepatitis B may have not been filed. However, the findings from this study suggest that a prospective study that collects data on infectious diseases among healthcare workers on a regular basis would be a realistic strategy in order to establish the actual incidence and prevalence of these diseases as well as their risk factors that could be targeted with specific interventions (Placidi et al., 2007; Mehtar, 2008; Talebi-Taher et al., 2010). Such a study should be part of an employee wellness program in line with the South African National Infection Control Policy and Strategy as enacted since 2007 with regard to employee health management (NDOH, 2007).

## 5. Conclusion

In conclusion, the most common occupational infection or hospital-acquired infection among healthcare workers in Limpopo province of South Africa was tuberculosis. It infected mainly nurses from the rural health district of Mopani. Younger age and being a nurse were significant risk factors associated with being infected early.
